# Dr. Arthur L. Reeve

**Published:** 1916-02

**Authors:** 


**Dr. Arthur L. Reeve,** L. I. College Hospital 1896, of Brooklyn, killed himself under peculiar circumstances Jan. 5. His wife was awakened by a shot and found her husband dead in bed. He was subject to nightmare, and only two nights previously had wandered about the house searching, in a dream, for a burglar, being awakened by bumping into a door. He kept a revolver under his pillow. No reason for intentional suicide existed. We mention this instance as a warning that weapons, kept for protection, may be a source of danger. Under ordinary conditions of civilized life, we believe that the general knowledge that one is not armed, is a protection in itself. A burglar or other assailant is not usually looking for trouble, and while he may shoot to anticipate being shot, he is not likely to do so if he knows that his victim is unarmed.

